# Disorders of Sleep

**Published:** 1907-07-06

**Authors:** 


### Diseases of Children.

### DISORDERS OF SLEEP.

He started up in bed with a piercing shriek, and shouted out, "The devil is here; take the knife away for he has cut her head off." His eyes were like saucers, his body trembled, and he sweated freely. Such is the vivid description of an attack of pavor nocturnus or night terrors in a boy aged five years.

These attacks come on in the early part of the night, an hour or two after going to sleep. The emotional distress is a prominent feature. It is a state of terror or mental confusion, perhaps a feeling of suffocation. The child is oblivious of its surroundings, does not recognise the attendant, and has hallucinations of vision, rarely auditory ones. Usually he falls asleep without recovering consciousness, in a few minutes to an hour, and next day has no remembrance, or merely a dim recollection of something horrible. There is rarely more than one attack a night. It may recur nightly for a time, at varying intervals, or quite irregularly. The hallucinations are almost always of the same nature.

In one variety the terror is subjective, more allied to nightmare, and possibly asphyxial in origin, the result of slowly developing carbonic-acid poisoning. Such attacks develop slowly, coming on an hour or more after going to sleep, and the feeling of suffocation, the mental confusion, hallucinations, amnesia, and muscular weakness are due to the asphyxia. The muscular weakness is shown by the inability to articulate clearly, so often present, and the clumsy movements. The susceptibility to this variety and the severity of the attacks are almost directly proportionate to the degree of nasal obstruction by adenoids and enlarged tonsils. Fright and hallucinations may occur in any disease leading to asphyxia, such as bronchitis, pneumonic affections, and faucial angina. Some attacks appear due to gastric or intestinal causes. If we accept the asphyxial view of the pathology, we must assume that in these cases the dyspnœa and sense of suffocation are due to reflex stimulation of the vagus.

In another variety, which may be called primary cerebral or idiopathic, the terror is objective, and is dependent on undue excitement of the cerebral cortex in a nervous child. Possibly alimentary toxins, circulating in the blood, induce the attack. A careful search must be made for any source of asphyxia. In these children there is often a predisposing factor in the shape of a neurotic or alcoholic parentage, a rheumatic diathesis, or a history of infantile convulsions. They show signs of neurasthenia; are nervous, irritable, impressionable, excitable, and often anæmic. They sleep lightly and restlessly, and are easily disturbed. The actual exciting cause may be the same as in the first variety of pavor; may be school work or excitement of any kind, dark rooms, tales of ghosts or gruesome pictures; or may be fever, or tuberculous meningitis, or injury to the head. In normal healthy children ghost tales and horrors do not induce an attack. Possibly they merely formulate the vision in the susceptible child.

In the cerebral type the pavor may be diurnal as well as nocturnal. An anæmic, dyspeptic boy, sixteen months old, had continual screaming attacks, of sudden onset and duration, with no loss of consciousness, and followed by trembling for about ten minutes. He got well. Pavor diurnus is rarely found independently of pavor nocturnus. It may be summed up as unaccountable terror and screaming while awake and perhaps in the middle of an enjoyable game.

Pavor is most common in the third and fourth years of life, the age at which adenoids produce most nasal obstruction. It remains fairly common up to the seventh year, and is rare after the age of ten. School life begins during this period, and, in addition, digestive disorders are liable to arise from dentition.

Nightmare, somnambulism, and teeth-grinding are affections allied to pavor. All may occur in the same child at different times. In nightmare there is a peculiar kind of dream, in which there is great distress, because of a feeling of inability to move and save oneself from a horrible or fatal situation. It is a terrifying dream, and causes the child to wake up frightened and excited, but the mind is clear, the attendants are recognised, and the occurrence, and often the details, of the dream are remembered next day. In pavor the child sees visions; in nightmare he dreams dreams.

Somnambulism is a type of nightmare. The term may be taken to include all varieties of motor action during sleep, and should not be limited to mere "walking." In its mildest form it is indicated by "talking in sleep." If more severe, the child will get out of bed, look for objects under it, open drawers and windows, walk downstairs, climb along dangerous parapets impossible to him while awake, and indeed perform almost any action, even dangerous ones, such as getting a knife and stabbing another child. The eyes are wide open, expressionless, and staring. Movements are deliberative and purposive, but intelligence is completely in abeyance and attendants are ignored. A bright light or a sharp order will send him back to bed. In one instance, a boy aged seven, there were hallucinations of "things crawling on the walls." Teeth-grinding is a habit due to similar causes. It is also apt to occur in neurotic and imbecile children. Other motor habits of the same type may be noted, such as scratching the stomach, turning over and kicking about, worrying himself, etc., in the expressive language of the mothers.

These sleep disorders are almost always symptomatic, and therefore the prognosis is excellent, except in so far that somnambulism may persist in those of neurotic ancestry, and may lead to fatal accident, and that the predisposing causes of pure cerebral pavor may be such as in later life give rise to epilepsy, insanity, migraine, tics, neurasthenia, and hysteria. In rare instances imbecility or serious brain disease may be present. Treatment is comparatively simple. Give an aperient, attend to the diet, and regulate the mode of life. Insist on efficient ventilation of the sleeping apartment. Permit the use of a night light, and let a nurse sleep in an adjacent room with the door open. Remove or cure causes of nasal obstruction. Allow no physical or mental strain. Limit school work to the morning hours. If there is any digestive mischief give a mixture of rhubarb and soda. To prevent recurrence at night give a dose of bromide or antipyrin at bedtime, or give antipyrin in doses of 1-4 grains, according to the age of the child, three times a day.

